# P-1215. Evaluation of Vancomycin AUC/MIC Ratio in Indian Patients with Enterococcal Infections: Impact on Clinical Outcomes and Nephrotoxicity

**DOI:** 10.1093/ofid/ofae631.1397

**Published:** 2025-01-29

**Authors:** Albin Sebastian, Jomel Raju, Maria Tom

**Affiliations:** St. Joseph's College of Pharmacy, Ponkunnam, Kerala, India; St. Joseph's College of Pharmacy, Cherthala, Pala, Kerala, India; St. Joseph's College of Pharmacy, Cherthala, Pala, Kerala, India

## Abstract

**Background:**

Vancomycin is commonly used to treat enterococcal infections, but its optimal dosage remains uncertain in Indian patient populations. This study aimed to investigate the relationship between vancomycin AUC/MIC ratio and clinical outcomes, as well as nephrotoxicity, in Indian patients with documented enterococcal infections. Understanding this relationship is crucial for optimizing treatment strategies and improving patient outcomes in this context.

**Methods:**

Data were collected retrospectively from medical records of 71 patients who received vancomycin with therapeutic drug monitoring at a single center in India between April 2015 and March 2022. Vancomycin AUC0–24 was calculated using a Bayesian technique, while MIC was determined using the VITEK 2 automated microbiology system. The AUC0–24/MIC ratio was computed for the initial 72 hours of therapy. Statistical analysis was conducted to assess the association between vancomycin AUC/MIC ratio and clinical outcomes, as well as nephrotoxicity.

**Results:**

The cohort comprised 71 participants meeting the inclusion criteria. Results indicated that a vancomycin AUC/MIC of ≥400 mg·h/L exhibited significant differences in clinical response (aHR: 0.49, 95% CI: 0.21–0.98; p = 0.044) and microbiological response (aHR: 0.41, 95% CI: 0.17–1.08; p = 0.039) compared to AUC/MIC < 400 mg·h/L. However, patients with AUC/MIC ≥400 mg·h/L had a higher incidence of nephrotoxicity compared to those with AUC/MIC < 400 mg·h/L (aHR: 3.41, 95% CI: 1.11–15.83; p = 0.033). Elevated vancomycin concentrations may lead to declining renal function. Moreover, declining renal function could result from factors such as failure to resolve the infection focus or co-administration of other antibiotics, potentially leading to higher AUC/MIC ratios. The study identified a target vancomycin AUC/MIC of ≥400 mg·h/L, suggesting its optimality for monitoring the treatment of enterococcal infections in the Indian context.

**Conclusion:**

In Indian patients with enterococcal infections, a vancomycin AUC/MIC of ≥400 mg·h/L is associated with improved clinical and microbiological responses but may increase the risk of nephrotoxicity. Therefore, careful monitoring of vancomycin dosage and renal function is essential to optimize treatment outcomes in this population.

**Disclosures:**

**All Authors**: No reported disclosures

